# Introduction of Non-Native Pollinators Can Lead to Trans-Continental Movement of Bee-Associated Fungi

**DOI:** 10.1371/journal.pone.0130560

**Published:** 2015-06-23

**Authors:** Shannon M. Hedtke, Eleanor J. Blitzer, Graham A. Montgomery, Bryan N. Danforth

**Affiliations:** 1 Department of Entomology, Cornell University, Ithaca, New York, United States of America; 2 Department of Ecology, Environment, and Evolution, La Trobe University, Bundoora, Victoria, Australia; Universidade de São Paulo, Faculdade de Filosofia Ciências e Letras de Ribeirão Preto, BRAZIL

## Abstract

Bees are essential pollinators for many flowering plants, including agriculturally important crops such as apple. As geographic ranges of bees or their host plants change as a result of human activities, we need to identify pathogens that could be transmitted among newly sympatric species to evaluate and anticipate their effects on bee communities. We used PCR screening and DNA sequencing to evaluate exposure to potentially disease-causing microorganisms in a pollinator of apple, the horned mason bee (*Osmia cornifrons*). We did not detect microsporidia, *Wolbachia*, or trypanosomes, which are common pathogens of bees, in any of the hundreds of mason bees screened. We did detect both pathogenic and apathogenic (saprophytic) fungal species in the genus *Ascosphaera* (chalkbrood), an unidentified species of *Aspergillus* fungus, and a strain of bacteria in the genus *Paenibacillus* that is probably apathogenic. We detected pathogenic fungal strains in asymptomatic adult bees that therefore may be carriers of disease. We demonstrate that fungi from the genus *Ascosphaera* have been transported to North America along with the bee from its native range in Japan, and that *O*. *cornifrons* is exposed to fungi previously only identified from nests of other related bee species. Further study will be required to quantify pathogenicity and health effects of these different microbial species on *O*. *cornifrons* and on closely-related native North American mason bees that may now be exposed to novel pathogens. A global perspective is required for pathogen research as geographic ranges of insects and microorganisms shift due to intentional or accidental introductions.

## Introduction

Estimates place the worldwide economic value of bee pollination well in the billions of dollars [1–4]. While the most widely-used bee managed for agricultural pollination is the European honey bee (*Apis mellifera*), other agriculturally-important managed bees include bumble bees (genus *Bombus*), alkali bees (*Nomia melanderi*), alfalfa leaf-cutter bees (*Megachile rotundata*), and mason bees (genus *Osmia*). The advent of Colony Collapse Disorder (CCD), resulting in the loss of one to two thirds of the managed European honey bee colonies in the United States [5–7], highlights the importance of early identification of pathogens in managed bees so that the impact and spread of disease can be controlled. While research has blossomed on honey bee and bumble bee diseases (e.g., [8–11]), pathogens in managed solitary bees, such as leaf-cutter and mason bees, have been relatively understudied. Our goal was to examine the frequency of exposure to potential pathogens across a geographic landscape in a managed solitary bee: the horned mason bee, *Osmia cornifrons*.

Solitary bees, including mason bees, are highly effective pollinators of early spring flowering trees [12]. Mason bees managed for fruit pollination include the red mason bee, *Osmia rufa*, in Europe [13–14]; the horned mason bee, *O*. *cornifrons*, in Japan [15]; and the blue orchard mason bee, *Osmia lignaria*, in the U.S. [16–17]. In the northeastern U.S., *O*. *lignaria* has been declining in abundance relative to other bees [18]. On the other hand, *O*. *cornifrons*, intentionally introduced to the U.S. from Japan in the 1970s [19], has been increasing in relative abundance [18]. It is now widespread across the eastern U.S. and in isolated locations in the western U.S. Whether the introduction of *O*. *cornifrons* has negatively impacted native *O*. *lignaria* is unclear. Both species are active in early spring, and *O*. *cornifrons* could outcompete *O*. *lignaria* for limited nest sites or floral resources such as pollen and nectar. As its range expands, *O*. *cornifrons* could introduce Japanese pathogens to populations of naive *O*. *lignaria*, disproportionately affecting the new hosts (as hypothesized for bumble bees [20]). To understand the impact of introduced pathogens to native bees, we first need to identify microbes that might cause disease and estimate their prevalence. We conducted our study on *O*. *cornifrons* in eastern apple orchards, as it is abundant and easily managed for experimental study.

**Fig 1:**
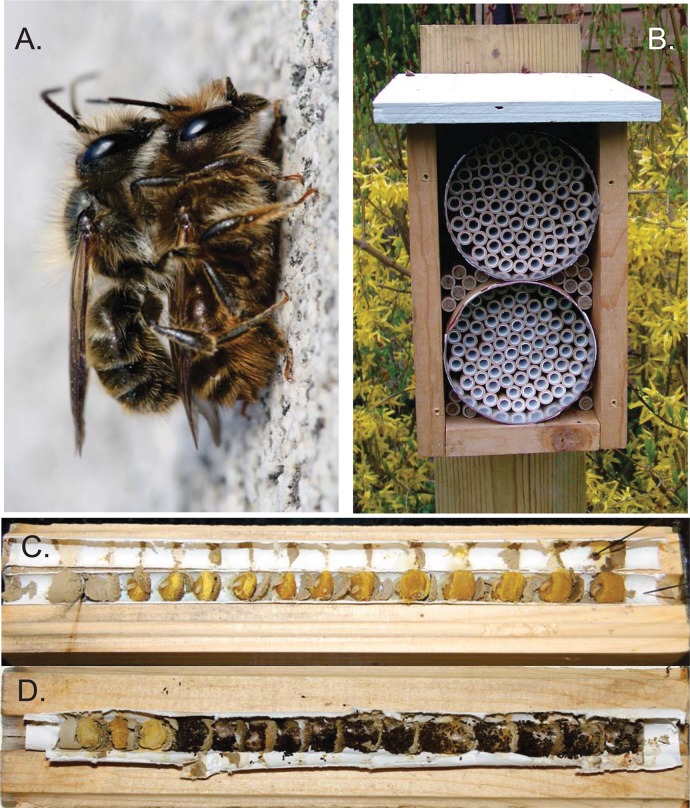
*Osmia cornifrons*. A) Mating pair of *O*. *cornifrons*; male above female. Photo credit: L. Russo, used with permission. B) Nest box used to provide shelter for mason bee trap nests. C) *O*. *cornifrons* nest opened shortly after nest closure; pollen provision masses with small larvae and eggs are visible; note mud partitions separating pollen provision masses. D) *O*. *cornifrons* nest opened after all larvae have completed feeding, defecated and spun cocoons (early fall); each cocoon contains one adult, diapausing bee. Nest entrances in figures C and D are to the left.

Stem-nesting bees, including mason bees and leaf-cutter bees, will readily take advantage of “trap nests”: artificial tubes made of wood or cardboard (Fig 1). Over the course of a few weeks, a mated female builds mud partitions to create separate cells within a trap nest or other hollow stem. Within each partitioned cell, she lays an egg on top of a ball of pollen and nectar [17,21–22]. Larvae consume this pollen provision, build a cocoon, pupate, and overwinter in the nest as diapausing adults (Fig 1; [17,21–22]). Thus, trap nests can be collected after nest completion, stored over winter, and placed in an orchard coincident with flowering the following season, where emerging bees will pollinate the fruit trees. The use of trap nests by mason bees allows us to comprehensively examine disease exposure in populations, since we can collect all individuals at a given location prior to spring emergence.

Larvae are exposed to disease when pollen provisions or mud partitions contain microorganisms. These microorganisms are carried and introduced to the nest by the mother, either because she is herself infected, or because she has inadvertently collected pathogens from the local environment during nest provisioning (as in pollen collected by honey bees [23]). For example, the disease chalkbrood occurs in stem-nesting bees when pollen provisions have been contaminated by spores of pathogenic fungi in the genus *Ascosphaera*. The spores are inadvertently consumed and germinate within the larval gut, sporulating under the larval cuticle [24–26]. Pathogenic *Ascosphaera* is often diagnosed by opening nests and looking for dead larvae with the mottled appearance caused by spores. However, not all *Ascosphaera* species are pathogenic. Saprophytic *Ascosphaera* consume pollen provisions, larval fecal pellets, the cocoon, or materials used to make the nest, with limited or uncertain indirect effects on bee fitness [27–31]. In Japanese nests of *O*. *cornifrons*, a number of *Ascosphaera* species have been detected [31]; whether these are pathogenic or apathogenic (saprophytic) is uncertain.

Other microorganisms are known to infect bees, but which of these may be found in *Osmia* is unknown. For example, certain species of the fungus *Aspergillus* can opportunistically cause disease in larvae and adult honey bees [32] and alkali bees [33]. The bacterium *Paenibacillus larvae* causes the disease foulbrood in European honey bees [34], while *Paenibacillus glucanolyticus* is associated with blackened bumble bee larvae under stress [35]. Pathogenic microsporidia include species in the genus *Nosema* that infect European and Asiatic honey bees and bumble bees [36–38] and the related *Antonospora scoticae* that infects the solitary, ground-nesting bee *Andrena scotica* [39]. Members of these groups of known disease-causing pathogens are obvious targets for examination across bee species.

The goal of this study was to examine possible pathogens in a solitary bee with the potential to be an important managed pollinator in apple orchards. We examined whether the frequency of exposure to microbes, or the effect of those microbes on fitness, differed across an agricultural landscape with variation in agrochemical application. We gathered *O*. *cornifrons* trap nests from three apple orchards with conventional pesticide management, from two organic apple orchards, and one personal residence, and tested hundreds of bees for microorganisms using PCR screens. Phylogenetic analysis of DNA sequence data of these PCR products indicate that this introduced bee species brought with it fungal species of *Ascosphaera* from its country of origin (Japan), and have been exposed to fungi previously only detected in other bee species, probably through shared floral resources. Our results open up the possibility that microorganisms from Japan could be introduced to North American native bees, particularly the congener *O*. *lignaria*. This study highlights the importance of a global perspective and rigorous screening of insects prior to release in new geographic areas.

## Materials and Methods

### Trap nests

We have established a source population of *Osmia cornifrons* within a residential area near the city of Ithaca in upstate New York, USA. Nests from this location were used to seed trap nests (Fig 1B) at five apple orchards and one residence within an 18-km radius. The owners of these private lands gave permission for this study, and we did not collect or harm any endangered or protected species. Orchards differed in management strategies for controlling pests, plant fungal pathogens, and weeds; conventionally managed orchards use inorganic insecticides, fungicides, and herbicides, while organic orchards use compounds derived from botanical or mineral sources. We have assigned each location a code to maintain confidentiality: CIC, CCI, and CCL for three conventionally managed orchards, OHG and OWH for two organic orchards, and RNV for the residential location (where no chemicals were applied). After spring activity was complete (June, 2011), we collected all capped nests (N = 153) and placed them in mesh bags to prevent attack by parasites or predators. These nests were maintained outside until after the bees had spun cocoons and entered diapause in the fall, when they were brought into the lab and maintained at ~4°C.

### Collection of nest data

Over the course of January through March 2012, each nest was opened and the nest contents were photographed (Fig 1D). Cells were counted (N = 771), and the condition of those without cocoons were noted (e.g., if they contained unconsumed pollen provisions, parasitoids, or dead larva). Each cocoon was removed from the nest with sterilized tweezers and opened. Species identification and sex was confirmed by examination under a dissecting microscope. Each live bee was weighed on a clean weigh boat and placed in a tube labeled by nest and position within the nest, starting with the cell closest to the back of the nest. Tubes were placed in a -80°C freezer until extraction. 626 individuals were collected in total, including 196 live females and 332 live males (S1 Table). This observed bias in sex ratio is common in mason bees [17].

### Nucleotide extraction

Nests from each population were randomly selected for PCR screening. All individuals from a nest were screened. We continued to screen nests until a minimum of 58 individuals were tested. This is a sample size that would detect microorganisms present at a frequency over 0.05 with an error rate of 0.05, under the conservative assumption of infinite population size, using the equation *n* = log β/log *p*, where β is the type II error rate and *p* is the proportion of animals in the population that do not have the microorganism [40]. For those sites where fewer than 58 total individuals were collected, all individuals were screened; at least 10 individuals are required to detect a microorganism at a prevalence of at least 25%, and 28 individuals are required to detect a microorganism at a prevalence of 10% or higher. The total sample size across populations was 326 mason bees, including live-frozen adults and dead larvae (S1 Table), as well as four honey bees for use as positive controls. DNA was extracted using a Chelex protocol modified from Boonham *et al*. [41] and Evison *et al*. [42]. Tubes were kept cold on ice. The metasoma of adult bees, or the entire body for dead larvae, was placed in a fresh tube in liquid nitrogen using sterilized instruments. 200 μl of cold, sterile water was placed in the tube on ice and the sample ground using a sterilized pestle attached to a cordless motor. 50 μl of this homogenate was aliquoted into a fresh tube (also on ice) with 50 μl of 50% chelating resin (Chelex100, BioRad). This mixture was vortexed briefly and heated to 100°C for 15 minutes. Tubes were then spun in a cold microcentrifuge for 5 minutes at 13,000g, and the supernatant containing DNA pipetted into a new tube for storage at -20°C.

### PCR and sequencing

The advantage of using PCR for screening is that it does not require culturing a particular microorganism for identification, it can be performed on DNA extractions that contain bee DNA (e.g., [43]), and the same extraction can be used to screen for multiple microorganisms. We selected primers from the literature to screen all 326 individuals for 6 microorganisms: fungi in the genus *Ascosphaera* and *Aspergillus*, bacteria in the genus *Paenibacillus* and *Wolbachia*, microsporidia, and trypanosomes (Table 1). A positive result was indicated by a single band visible after gel electrophoresis and ethidium bromide staining. We calculated the percentage of males and females from each population that tested positive for each microorganism. To test the null hypothesis that positive individuals and negative individuals have the same mean weight, we performed a Welch's t-test for each site individually and across all sites using R v.3.1.1 for Linux [44]. For all positives (*Ascosphaera*, n = 129; *Aspergillus*, n = 129; *Paenibacillus*, n = 11), we performed Sanger sequencing in both directions at the Biotechnology Resource Center at Cornell University. For those PCR amplicons that were successfully sequenced (*Ascosphaera*, n = 85; *Aspergillus*, n = 28; *Paenibacillus*, n = 8), we trimmed the primer sequence and confirmed that the expected microbial genus had been amplified by a blastn search of the sequence to the NCBI GenBank database [45]. A best hit to another genus would have indicated unspecific PCR amplification. We used a DNA sample extracted from a European honey bee (*Apis mellifera*) to confirm that PCR amplification and sequencing conditions were appropriate for the primers selected for microsporidia and trypanosomatids, as none of the *Osmia* tested were positive for these microorganisms (see Results). Sequences have been deposited into the NCBI nucleotide sequence database (Table 1).

**Table 1:** Microbes targeted for screening *Osmia cornifrons*.

Target	PCR primers	Annealing temp (°C)	Alignment length (bp)	GenBank accession nos.
*Ascosphaera* spp.: internal transcribed spacer 1 (partial), 5.8S, internal transcribed spacer 2 (partial)	AscoAll-F, AscoAll-R [43]	62	466	KP340870–KP340896
*Aspergillus* spp.: 28S rRNA subunit (partial)	AF4, AR1 [46]	54	222	KP340862–KP340869
Microsporidia: small rRNA subunit (partial)	MicroF, 1492N [47]	54	N/A	N/A
*Paenibacillus* spp.: 16S rRNA subunit (partial)	AF1f, AF2 [48]	58	146	KP340861
Trypanosomes: small rRNA subunit (partial)	TrypanF1, TrypanR1 [49]	58	N/A	N/A
*Wolbachia* spp.: cytochrome oxidase A (partial)	coxF1, coxR1 [50]	54	N/A	N/A

### Phylogenetic analyses

In August, 2014, we downloaded the following sequences from NCBI’s GenBank non-redundant nucleotide sequence database: (1) Internal transcribed spacer 1 (ITS-1), 5.8S ribosomal DNA, and internal transcribed spacer 2 (ITS-2) for all available *Ascosphaera* species plus two outgroups (per [31,51]); (2) 28S ribosomal DNA for select *Aspergillus* species within clades that contain both bee disease-causing strains (from [32]), species that are the best BLAST hit to our sequences [45], plus two outgroups [52]; (3) one 16S ribosomal DNA sequence from each *Paenibacillus* species available. Sequences from GenBank and this study were aligned by eye using Mesquite v.2.75 [53], trimmed so that downloaded and new sequences were the same length, and identical sequences removed. The maximum likelihood estimate for each of the three alignments was estimated using RAx-ML-AVX v.8.1.2 for Linux [54], with 20 search replicates under the GTRCAT model (commands:-# 20-m GTRCAT). Bootstrap support was determined using the long search method with 1000 search replicates (commands:-b 5 -# 1000-m GTRCAT).

We selected a model of sequence evolution using jModelTest v.2.1.6 [55]. When constrained to the smallest number of models available in the program (n = 24), the SYM+I+G model of sequence evolution was selected as the best model for the *Ascosphaera* alignment under the sample-size corrected Akaike information criterion (AICc [56]), Bayesian information criterion (BIC [57]), and decision theory (DT [58]) methods for model selection. The best-fit model for both *Aspergillus* and *Paenibacillus* alignments was either K80+I+G (AICc) or GTR+I+G (BIC and DT). We estimated the maximum likelihood tree via PhyML v.3.0 for Linux [59] using model averaging, and under the best-fit model, with bootstrap proportions estimated using 1000 replicates. We used MrBayes v.3.2.2 [60] to compute posterior probabilities of bipartitions; four runs of 10,000,000 generations each with a sample frequency of 1000 resulted in 10,000 sampled trees/parameters. To avoid getting trapped in a region of tree space with excessively long branch lengths [61], we set the branch length prior to represent an exponential distribution with an expected mean of 0.01, rather than the default of 0.10 (command: prset brlenspr = Unconstrained:Exp(100)). Tracer v.1.6 [62] was used to examine individual runs for stationarity, to ensure convergence among runs for all parameter estimates by comparing posterior marginal distributions, and to set a burn-in that would result in ESS values well over 200 for all parameter values. Topological convergence among runs was assessed by requiring the deviations in split frequency (as estimated by MrBayes) to be less than 0.005, and by visually comparing splits between pairs of runs using the web-portal for AWTY [63]. For analyses of all three alignments, runs appeared to reach stationarity and convergence very rapidly, in less than 50,000 generations. Based on this observation, burn-in was set at 10%, leaving 36,000 samples (representing 4 x 9 million generations) for posterior probability calculations. Figures were produced using the R package APE v.3.1–4 [64].

## Results

### Nest provisioning and offspring survival

We observed differences among sampling locations in the number of nests collected, the number of cells per nest, the number of live bees within the nest, and the sex ratio of live bees (Table 2). Many cells at each location were empty or contained only pollen provisions, dead larva, dead adults, or other insects such as parasitoids (S1 Table). A Welch's t-test comparing the mean number of cells per nest at organic orchards versus conventional orchards suggested these means were significantly different (p < 0.005). However, differences in the number of live bees per nest and the M:F sex ratios at each location were not correlated with orchard management (null hypothesis that means were the same not rejected at p = 0.08619 and p = 0.2955, respectively). Note that the number of replicates within each category (two organic versus three conventional orchards) is quite low, and thus these results have uncertain ecological causes.

**Table 2:** Summary statistics on diapaused adult bees from cells of *Osmia cornifrons* nests collected at one residence (R) and five orchards with organic (O) or conventional (C) management practices.

Site code	# nests	# cells	Mean # cells/nest	Mean # live adults/nest	# live females	# live males	Sex ratio M:F
RNV	11	59	5.36	3.45	10	28	2.8
OHG	37	262	7.08	4.24	57	100	1.75
OWH	40	306	7.65	6.05	96	142	1.48
CCL	20	65	3.25	2.3	15	31	2.07
CIC	11	49	4.45	3.55	15	24	1.6
CCI	8*	30	3.75	1.12	2	7	3.5
Mean	21.2	128.5	5.26	3.46	33	55.8	2.2

* Some nests were destroyed when a farm vehicle hit the nesting box.

### Detection of microorganisms in *Osmia cornifrons* nests

Percentages of each microorganism detected by PCR screening are reported in Table 2 (see also S1 Table). Fungi in the genus *Ascosphaera* and *Aspergillus* have a strong association with *O*. *cornifrons* in upstate New York (Table 3). Bacteria in the genus *Paenibacillus* were detected at relatively low frequency at only a couple of sites (Table 3). Bees testing positive for more than one of these microbes—usually *Ascosphaera* and *Aspergillus*—were relatively common across sites. No positives were observed for microsporidia, trypanosomes, or *Wolbachia* in any of the 326 individuals tested. The efficacy of the first two markers was confirmed using DNA extraction, amplification, and sequencing from a European honey bee that was positive for sequences with best blast hits to *Nosema ceranae* and to trypanosomes.

**Table 3:** Counts and mean weight per site of *Osmia cornifrons* that tested positive (+) or negative (-) based on PCR screens for *Ascosphaera* (Asc), *Aspergillus* (Asp), *Paenibacillus* (Pae), and multiple microbes (mul).

Site code	# inds tested	% -	% Asc+	% Asp+	% Pae+	% mul+	mean weight (mg)^a^	mean weight - (mg)	mean weight + (mg)
**Females**									
RNV	10	60.0	10.0	30.0	0	0	52.7	49.4	57.7
OHG	33	30.3	51.5	48.5	12.1	39.4	49.2	45.9	50.6
OWH	28	82.1	10.7	7.1	0	0	59.5	58.3	64.7
CCL	16	75.0	18.8	12.5	0	6.3	41.0	43.6	33.2
CIC	18	50.0	22.2	38.9	0	11.1	48.5	54.7	42.3*
CCI	2	50.0	50.0	0	0	0	37.3	34.0	40.5
Mean	17.8	57.9	27.2	22.8	2.0	56.8	48.0	47.7	48.1
**Males**									
RNV	28	28.6	57.1	42.9	3.6	28.6	30.3	31.8	29.7
OHG	66	15.2	62.1	72.7	7.6	54.5	35.2	35.4	35.2*
OWH	42	54.8	33.3	19.0	0	7.1	36.5	37.1	35.9
CCL	30	50.0	20.0	43.3	0	13.3	32.3	35.9	28.6
CIC	20	50.0	25.0	40.0	0	15.0	33.9	37.6	30.2
CCI	7	28.6	71.4	14.3	0	14.3	23.5	26.3	22.4
Mean	32.1	37.9	44.8	38.7	1.9	22.1	32.0	34.0	30.3*
**Dead larvae/adults**									
RNV	4	100	0	0	0	0	NA	NA	NA
OHG	9	33.3	55.6	55.6	0	44.4	NA	NA	NA
OWH	6	16.7	83.3	33.3	0	33.3	NA	NA	NA
CCL	4	100	0	0	0	0	NA	NA	NA
CIC	1	0	100	100	0	100	NA	NA	NA
CCI	2	0	100	50	100	100	NA	NA	NA
Mean	4.3	41.7	56.5	39.8	8.3	46.3	NA	NA	NA

^a^ Mean values of the weight in milligrams (mg) of live bees that were then screened for the presence of microbes (mean weight), of only those bees testing negative (mean weight -), and of only those bees testing positive for one or more microorganism(s) (mean weight +).

* Mean weight of bees testing positive versus negative were significantly different based on a Welch's t-test at p < 0.05.

On the basis of a Welch's t-test comparing the means of bees testing positive and negative across all sites, males that tested positive tended to have a lower weight compared to males that tested negative (Table 3). At CIC, differences in mean weight of females testing positive and negative were significantly different (p = 0.04189), and at OHG, the weight of males testing positive and negative were significantly different (p = 0.005413). However, sample sizes per sex per site are low, and a negative result for the pathogens tested here does not ensure that bees are disease-free; other infectious organisms or viruses may be present but not detected. Finally, we were able to amplify and sequence markers for microbes extracted from several dead larvae and dead adult bees found in nests (Table 3).

### Phylogenetic analyses suggest identities for microorganisms detected

**Fig 2:**
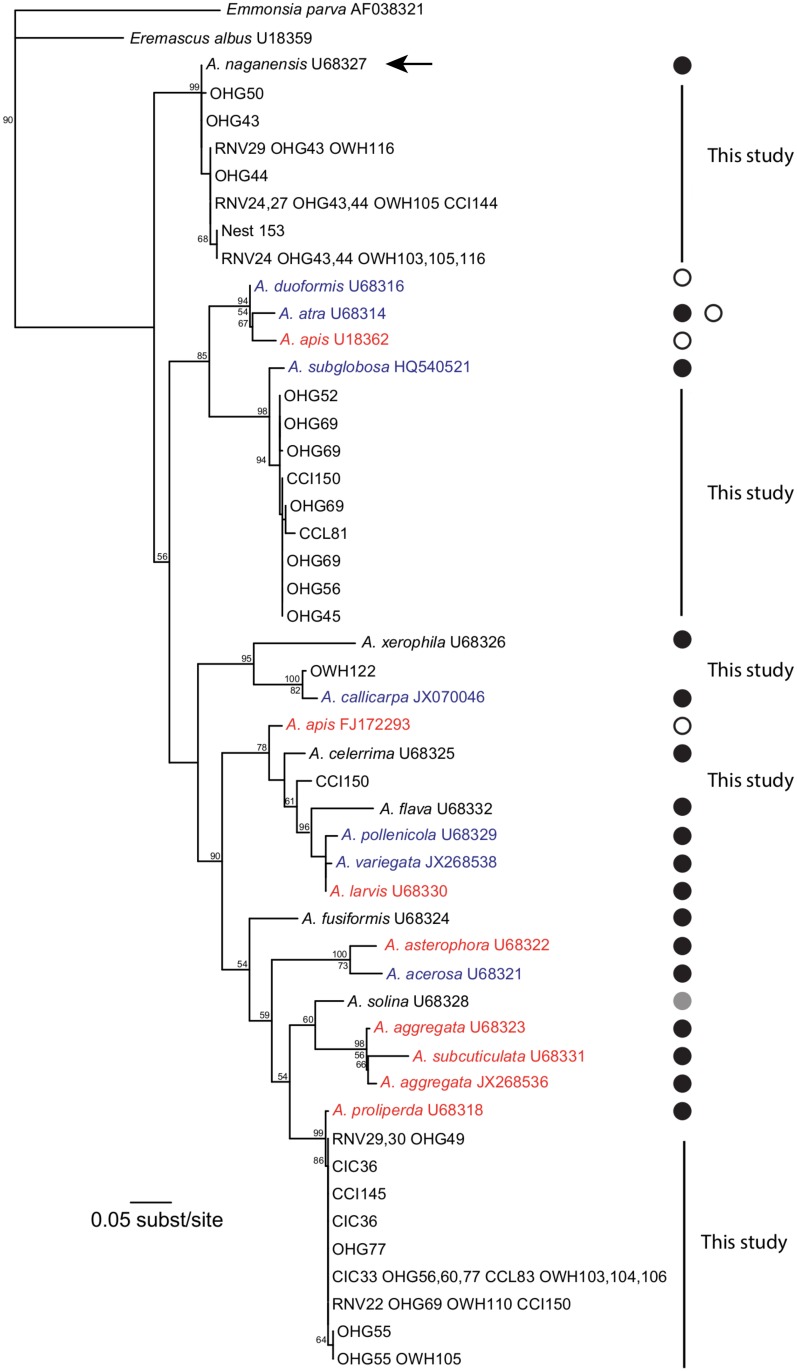
Maximum-likelihood estimate of *Ascosphaera* species based on ITS-1, 5.8S, and ITS-2 DNA sequences. Numbers above nodes represent bootstrap proportions; numbers below nodes represent posterior probabilities. Values below 50% have been removed to enhance readability and interpretation. Sequences from this study are indicated by sample location code and nest number; sequences from dead larvae have an asterisk (*). Colors indicate fungal life history (and possible pathogenicity); red: pathogenic, blue: saprophytic, black: unknown. Circles indicate host family; black circles: found in nests of Megachilidae, gray circles: found in nests of Colletidae, open circles: found in colonies of Apidae. Host and pathogenicity from references [24,28–31,65–67]. *Ascosphaera naganensis*, a species whose holotype was collected from *Osmia cornifrons* in its native range in Japan, is indicated by an arrow.

**Fig 3:**
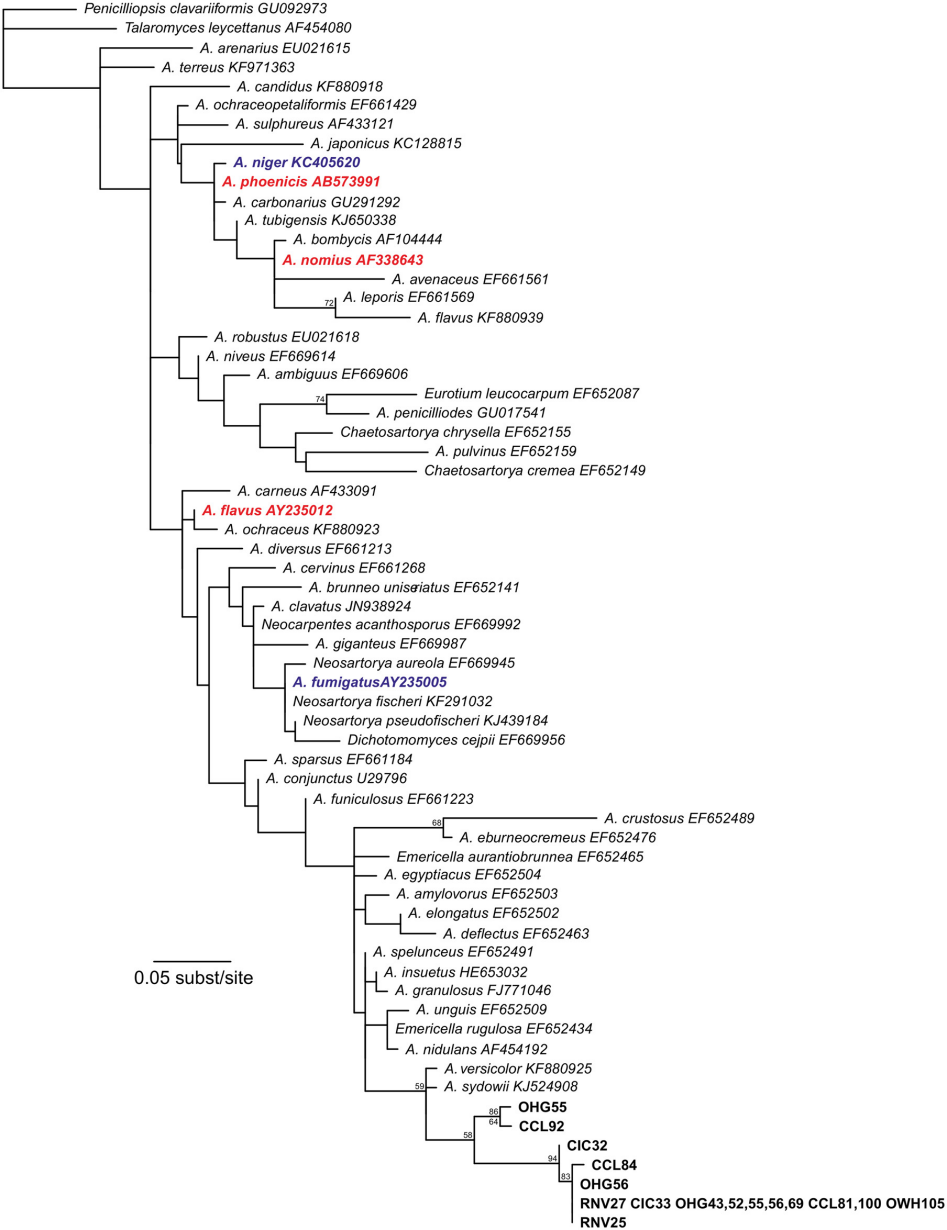
Maximum-likelihood estimate of *Aspergillus* species based on 28S DNA sequence data. Numbers above nodes represent bootstrap proportions; numbers below nodes represent posterior probabilities. Values below 50% have been removed to enhance readability and interpretation. Sequences from this study are indicated by sample location code and nest number. Colors indicate pathogenicity as tested in honey bees [32]; red: pathogenic, blue: apathogenic, black: not tested.

Posterior probabilities for bipartitions had a surprising tendency to be lower than bootstrap proportions, which were on the whole quite low. This is driven by the relative lack of variation in the sequence data; markers were chosen for their specificity to a particular microorganism, and not to maximize phylogenetic signal. The maximum likelihood estimates across the approaches used (PhyML, RAxML, and GARLI) and the Bayesian consensus tree did contain compatible clades (Figs 2–3; S1 Fig; S1–S3 Files). Given these caveats, we would tentatively assign several strains detected to the species *Ascosphaera naganensis*, *Ascosphaera proliperda*, and *Ascosphaera subglobosa*, one strain as either *Ascosphaera callicarpa* or a very close relative, and leave the remainder as unidentified. We were able to sequence *Ascosphaera* from some of the dead larvae found in nests; strains sequenced from these were closely related to *A*. *proliperda*, *A*. *subglobosa*, *A*. *naganensis*, and an unknown strain in a clade that includes the pathogen *Ascosphaera larvis*.

## Discussion

We screened hundreds of agriculturally-important mason bees from orchards that range in pesticide application to determine the frequency and identity of potential pathogens. Prior to their emergence from the nest, larvae and diapausing adults have been exposed to pathogenic and apathogenic fungi in the genus *Ascosphaera*, to a novel fungal species of *Aspergillus*, and to (probably benign) bacteria in the genus *Paenibacillus* (Table 3). *Osmia cornifrons* from the northeastern U.S. are associated with microbes that are likely to have their geographic origin in Japan, as well as fungi shared with bee species in the families Megachilidae and Apidae. Differences among sites in the number of offspring collected in trap nests and the average weight of those offspring do not appear to be correlated with population pathogen load. This study highlights the complexity of interactions among communities of bees and their pathogens.

### Microbial identification and origin

#### Fungal species of *Ascosphaera*

Infection of larvae with *Ascosphaera*, the causative agent of chalkbrood, occurs after they ingest fungal spores on the pollen and nectar ball provisioned by their mother [25–26]. Their mother either carried fungal spores from her birth nest, or gathered them from flowers or soil. Saprophytic *Ascosphaera* can be found in larval fecal pellets, mud partitions, or cocoons. Our screens were not designed to distinguish among microbes that infect and could cause disease and microbes found on the exterior of the bee. We rely on DNA sequence data and phylogenetic analysis to make tentative identifications of fungal species.

*Ascosphaera naganensis* was common across populations (n = 35; Fig 2). The holotype of this species is from *O*. *cornifrons* nests in Honshu, Japan [31], which is also the geographic origin of the introduced bees [19]. Without extensive screening of northeastern bee populations prior the introduction of *O*. *cornifrons*, we cannot conclusively rule out the possibility that these exotic mason bees have become associated with an American strain of *A*. *naganensis*. However, chalkbrood was observed in *O*. *cornifrons* nests prior to their initial release. Infected larvae were destroyed, and no infected larvae were reported the following season [19]. We detected both pathogenic and saprophytic fungi associated with asymptomatic carriers. The geographic origin of the bee, the association of this fungal holotype with bees from the same region, the similarity of DNA sequence data collected in Japan to those we collected, and the ability of *Ascosphaera* fungal spores to escape visual detection strongly suggests that *A*. *naganensis* has been carried across the globe along with the bee despite precautions.

In asymptomatic adult bees (n = 29), dead larvae (n = 7), and dead bees (n = 3), we detected fungi closely related to a pathogen, *Ascosphaera proliperda* (Fig 2). This fungus has previously been identified as a disease agent of chalkbrood in another megachilid bee, *Megachile centuncularis*, from Europe [65], but has not been previously reported in Japan. *Megachile centuncularis* is an introduced species now widespread in the northern U.S. and Canada [68–69], is polylectic, and is active in early June [70]. Exposure to *A*. *proliperda* may have occurred in the U.S. after *O*. *cornifrons* was introduced.

Another species detected, *Ascosphaera subglobosa* (Fig 2), is also likely to have been introduced after *O*. *cornifrons* establishment in the U.S. *Ascosphaera subglobosa* has previously been identified in nests of *Megachile rotundata* from the United States and Canada, and is considered a saprophyte with no effect on bee fitness [30]. Although most of our sequences were amplified from adult bees (n = 8), we did sequence this strain in one dead larva. However, this does not necessarily implicate *A*. *subglobosa* as the cause of mortality—multiple strains of *Ascosphaera* can be present in a nest (e.g., [31]), and our screens were not designed to distinguish either the cause of mortality or the presence of multiple strains.

Finally, we detected two additional species not previously reported in *Osmia*: a strain closely related to *Ascosphaera callicarpa* (Fig 2), previously identified from larval fecal pellets of *Chelostoma florisomne* from Europe [66], and a strain which shares a common ancestor with *Ascosphaera larvis* and *Ascosphaera apis*, causative agents of chalkbrood in leaf-cutter bees [28] and honey bees [71], respectively. Additional research on *Ascosphaera* across bee species, and across the globe, will be necessary to determine whether these fungi had their origins in Japan or in America.

The mechanisms for host-switching in *Ascosphaera* have not been conclusively determined. Fungal spores are likely to be transmitted among species through shared resources in the same manner that they are transmitted among individuals of the same species. Bee species with overlapping geographic distributions and emergence times visit the same flowers, and these flowers could serve as vectors for fungi and other pathogenic organisms [23,72].

#### An unknown species of fungus in the genus *Aspergillus*

We also detected fungi in the genus *Aspergillus* associated with *O*. *cornifons* in our populations. Unlike *Ascosphaera*, *Aspergillus* fungi are not obligately associated with insects, and only some of these cause disease. *Aspergillus* species that have caused mortality in adult and larval honey bees, including *A*. *phoenicis*, *A*. *nomius*, and *A*. *flavus* [32], were not closely related to the strains detected in our study (Fig 3). Indeed, our *Aspergillus* sequences cluster as a well-supported clade divergent from any known sequences, making it impossible to speculate on species identification. As they were found across our study sites, this species may be a common soil microbe in upstate New York. The most closely-related sister species, *A*. *versicolor* and *A*. *sydowii*, can be opportunistic pathogens that cause aspergillosis in humans (e.g., [73]) and fan corals [74]. Thus, while our study demonstrates that this fungus is present within nests, the effects on bee fitness will require additional tests.

#### Detection of *Paenibacillus* bacteria

There is no evidence that the other microbe detected in our study, bacteria in the genus *Paenibacillus*, is pathogenic to bees. While the region used for screening was not particularly variable, our 16S sequences were all identical to each other and identical to sequences from GenBank of two species: *Paenibacillus terrae*, a xylanase-producing bacterium being studied for industry [75], and *Paenibacillus polymyxa*, a strain that fixes nitrogen and is widely used in agriculture [76]. *Paenibacillus larvae*, the chitin-degrading bacterium responsible for foulbrood [77], is not closely related to either species (S1 Fig; S3 File).

#### Undetected microbial species

While other bee species have been discovered infected with microsporidia, trypanosomes, and *Wolbachia* [8,39,42,49], we failed to detect them in any of the 326 *O*. *cornifrons* screened (Table 3). Experimental error cannot be ruled out, although both microsporidia (*Nosema apis)* and trypanosomes (*Crithidia mellificae*) were successfully detected in our control sample, the European honey bee. *Osmia cornifrons* may be more resistant to infection by microorganisms within these clades of known pathogens due to behaviors that reduce exposure, to effective immune system response at time of exposure, or because no pathogens within these clades have evolved specificity to *O*. *cornifrons*. That said, *Wolbachia*, *Nosema*, and *Crithidia* have been detected in several other osmiine bees [78–79]. Alternatively, the bottleneck that most likely occurred when *O*. *cornifrons* was introduced into the U.S. may have allowed for pathogen escape: only microorganisms associated with that subsample of bees would be present in contemporary populations. Teasing out these possibilities requires screening nests collected from populations across Japan, as well as from other megachilids collected in the eastern U.S.

### Mortality and fitness in the horned mason bee

Females that provisioned collected nests were from the same source population. However, we observed differences among sites in characteristics that may be indicators of female and population fitness: the number of nests established, the number of cells with diapaused adults per nest (i.e., offspring per female), and mean body weight of diapaused adults (Table 2, S1 Table). Based on the frequency of observed microbes across populations (Table 3), variation among sites does not appear to be related to differences in pathogen exposure. Environmental characteristics may better explain these observed differences. Low establishment success at the residential site (RNV), for example, may be due to resource availability, since there were few flowering trees compared to the abundance of flowers in orchards, or could be due to differences in nest-site availability, with bees preferring nesting sites elsewhere in the residential area to the nesting box provided. Among orchard sites, factors such as microclimate variation, position of nests relative to prevailing winds, or chemical environment (conventional agrochemical use vs. organic chemicals) are likely to play important roles in bee fitness. *Osmia* larvae could be exposed to agrochemicals in pollen provisions, including pesticides, fungicides and herbicides. Different bee species vary in their response to chemical exposure [80–81], but one effect observed is reduced immune system response (e.g., [82]). The impact of agrochemicals thus complicates predicting the response of any particular species to pathogen exposure. The effects of microbial exposure on bee population fitness requires careful examination of interactions among landscape features, chemical environment, behavior, and immune system function.

## Conclusions

*Osmia cornifrons* is not native to U.S., and evidence suggests that fungi from Japan continue to be associated with these bees. An open question is whether microbes or viruses originating from Japan have inadvertently been introduced to native bees. The blue orchard mason bee, *Osmia lignaria*, is a native pollinator [16–17] whose range overlaps with the introduced *O*. *cornifrons*. Relative to other bees, *O*. *lignaria* have been declining in number in the northeastern U.S. [18] for unknown reasons. Furthermore, attempts to re-establish *O*. *lignaria* in residential areas in upstate New York by seeding sites with commercially-obtained, diapausing adults have not been successful (M. Park, M. Centrella, personal communications). Future studies on these bees will examine whether the decline of *O*. *lignaria* is associated with sympatry with *O*. *cornifrons* or is independent of the introduced species. Where the species are sympatric, competition for resources or differential response to microorganisms or viruses could be contributing to poor fitness in native populations.

This study contributes to the growing evidence that pathogens can be transmitted among agriculturally-important bee species (e.g., [23,72,83]). We cannot examine a single species of bee and hope to track its epidemiological history. Rather, we must consider pathogens across bee species, and, given intentional and unintentional range expansions of non-native species, across the globe.

## Supporting Information

S1 Fig Maximum-likelihood estimate of *Paenibacillus* species based on a fragment of 16S DNA sequence. Numbers above nodes represent bootstrap proportions; numbers below nodes represent posterior probabilities.
(EPS)

S1 File Trees in Newick format from maximum likelihood and Bayesian analyses of ITS-1, 5.8S, and ITS-2 DNA sequence data from fungi in the genus *Ascosphaera*. (TXT)

S2 File Trees in Newick format from maximum likelihood and Bayesian analyses of 28S sequence data from *Aspergillus*. (TXT)

S3 File Trees in Newick format from maximum likelihood and Bayesian analyses of 16S sequence data from *Paenibacillus*. (TXT)

S1 Table Nest position, sex, weight, and whether individuals tested positive for 3 microbes in 6 populations of *Osmia cornifrons*. (XLS)

## References

[1] Southwick EE, Southwick L Jr (1992) Estimating the economic value of honey bees (Hymenoptera: Apidae) as agricultural pollinators in the United States. J Econ Entomol 85: 621–633.

[2] Morse RA, Calderone NW (2000) The value of honey bees as pollinators of U.S. crops in 2000. Bee Culture 128: 1–15.

[3] Gallai N, Salles J-M, Settele J, Vaissière BE (2008) Economic valuation of the vulnerability of world agriculture confronted with pollinator decline. Ecol Econ 68: 810–821. 10.1016/j.ecolecon.2008.06.014

[4] Calderone NW (2012) Insect pollinated crops, insect pollinators and US agriculture: trend analysis of aggregate data for the period 1992–2009. PLOS ONE 7: e37235 10.1371/journal.pone.0037235 22629374 PMC3358326

[5] Stankus T (2008) A review and bibliography of the literature of honey bee colony collapse disorder: a poorly understood epidemic that clearly threatens the successful pollination of billions of dollars of crops in America. J Agr Food Inf 9: 115–142. 10.1080/10496500802173939

[6] vanEngelsdorp D, Hayes J Jr., Underwood RM, Pettis J (2008) A survey of honey bee colony losses in the U.S., Fall 2007 to Spring 2008. PLOS ONE 3: e4071 10.1371/journal.pone.0004071 19115015 PMC2606032

[7] vanEngelsdorp D, Evans JD, Saegerman C, Mullin C, Haubruge E, Nguyen BK, et al (2009) Colony Collapse Disorder: a descriptive study. PLOS ONE 4: e6481 10.1371/journal.pone.0006481 19649264 PMC2715894

[8] Runckel C, Flenniken ML, Engel JC, Ganem D, Andino R, DeRisi JL (2011) Temporal analysis of the honey bee microbiome reveals four novel viruses and seasonal prevalence of known viruses, *Nosema*, and *Crithidia* . PLOS One 6: e20656 10.1371/journal.pone.0020656 21687739 PMC3110205

[9] Martin SJ, Highfield AC, Brettell L, Villalobos EM, Budge GE, Powell M, et al (2012) Global honey bee viral landscape altered by a parasitic mite. Science 336: 1304–1306. 10.1126/science.1220941 22679096

[10] Graystock P, Yates K, Evison SEF, Darvill B, Goulson D, Hughes WOH (2013) The Trojan hives: pollinator pathogens, imported and distributed in bumblebee colonies. J Appl Ecol 50: 1207–1215. 10.1111/1365-2664.12134

[11] Fürst MA, McMahon DP, Osborne JL, Paxton RJ, Brown MJF (2014) Disease associations between honeybees and bumblebees as a threat to wild pollinators. Nature 506: 364–366. 10.1038/nature12977 24553241 PMC3985068

[12] Park MG, Orr MC, Danforth BND, Hall C (2010) The role of native bees in apple pollination. NY Fruit Quart 18: 21–25.

[13] Free JB, Williams IH (1970) Preliminary investigations on the occupation of artificial nests by *Osmia rufa* L. (Hymenoptera, Megachilidae). J Appl Ecol 73: 559–566.

[14] Holm SN (1973) *Osmia rufa* L. (Hymenoptera) as a pollinator of plants in greenhouses. Entomol Scand 4: 217–223.

[15] Yamada M, Oyama N, Sekita N, Shirasaki S, Tsugawa C (1971) Preservation and utilization of natural enemies and useful insects in apple orchards III. The ecology of the megachilid bee, *Osmia cornifrons* (Radoszkowski) and its utilization for apple pollination. Bull Aomori Apple Exp Sta No 15: 1–80.

[16] Torchio PE (1976) Use of *Osmia lignaria* Say (Hymenoptera: Apoidea, Megachilidae) as a pollinator in an apple and prune orchard. J Kansas Entomol Soc 49: 475–482.

[17] Bosch J, Kemp WP (2000) Development and emergence of the orchard pollinator *Osmia lignaria* (Hymenoptera: Megachilidae). Environ Entomol 29: 8–13. 10.1603/0046-225X-29.1.8

[18] Bartomeus I, Ascher JS, Gibbs J, Danforth BN, Wagner DL, Hedtke SM, et al (2013) Historical changes in northeastern United States bee pollinators related to shared ecological traits. Proc Natl Acad Sci USA 110: 4656–4660. 10.1073/pnas.1218503110 23487768 PMC3606985

[19] Batra SWT (1978) *Osmia cornifrons* and *Pithitis smaragdula*, two Asian bees introduced into the United States for crop pollination. Proc IVth Int Symp on Pollination Md Agric Exp Sta Spec Misc Publ 1: 307–312.

[20] Cameron SA, Lozier JD, Strange JP, Koch JB, Cordes N, Solter LF, et al (2011) Patterns of widespread decline in North American bumble bees. Proc Natl Acad Sci USA 108: 662–667. 10.1073/pnas.1014743108 21199943 PMC3021065

[21] Bosch J, Kemp WP (2001) How to manage the Blue Orchard Bee as an Orchard Pollinator. Beltsville, MD: Sustainable Agriculture Network 88 p.

[22] Bosch J, Sgolastra F, Kemp WP (2008) Life cycle ecophysiology of *Osmia* mason bees used as crop pollinators In: James R, Pitts-Singer T, editors. Bee Pollination in Agricultural Ecosystems. Oxford, New York: Oxford University Press pp. 83–104.

[23] Singh R, Levitt AL, Rajotte EG, Holmes EC, Ostiguy N, vanEngelsdorp D, et al (2010) RNA viruses in hymenopteran pollinators: evidence of inter-taxa virus transmission via pollen and potential impact on non-*Apis* hymenopteran species. PLOS ONE 5: e14357 10.1371/journal.pone.0014357 21203504 PMC3008715

[24] Skou JP (1975) Two new species of *Ascosphaera* and notes on the conidial state of *Bettsia alvei* . Friesia 11: 62–74.

[25] Stephen WP, Vandenberg JD, Fichter BL (1981) Etiology and epizootiology of chalkbrood in the alfalfa leafcutting bee *Megachile rotundata*, with notes on *Ascosphaera* species. Oreg State Univ Agr Exp Station Bull 653: 1–10.

[26] McManus WR, Youssef NN (1984) Life cycle of the chalk brood fungus, *Ascosphaera aggregata*, in the alfalfa leafcutting bee, *Megachile rotundata*, and its associated symptomatology. Mycologia 76: 830–842.

[27] Skou JP (1985) Notes on the habitats, morphology and taxonomy of spore cyst fungi (Ascosphaerales). Apiacta 4: 105–109.

[28] Bissett J (1988) Contribution toward a monograph of the genus *Ascosphaera* . Can J Bot 66: 2541–2560. 10.1139/b88-346

[29] Anderson DL, Gibson NL (1998) New species and isolates of spore-cyst fungi (Plectomycetes: Ascosphaerales) from Australia. Aust Syst Bot 11: 53–72. 10.1071/sb96026

[30] Wynns AA, Jensen AB, Eilenberg J, James R (2012) *Ascosphaera subglobosa*, a new spore cyst fungus from North America associated with the solitary bee *Megachile rotundata* . Mycologia 104: 108–114. 10.3852/10-047 21828215

[31] Skou JP (1988) Japanese species of *Ascosphaera* . Mycotaxon 31: 173–190.

[32] Foley K, Fazio G, Jensen AB, Hughes WO (2012) Nutritional limitation and resistance to opportunistic *Aspergillus* parasites in honey bee larvae. J Invertebr Pathol 111: 68–73. 10.1016/j.jip.2012.06.006 22750047

[33] Batra SWT, Bohart GE (1969) Alkali bees: response of adults to pathogenic fungi in brood cells. Science 8: 607. 10.1126/science.165.3893.607 17770861

[34] Genersch E, Forsgren E, Pentikäinen J, Ashiralieva A, Rauch S, Kilwinski J, et al (2006) Reclassification of *Paenibacillus larvae* subsp. *pulvifaciens* and *Paenibacillus larvae* subsp. *larvae* as *Paenibacillus larvae* without subspecies differentiation. Int J Syst Evol Microbiol 56: 501–511. 16514018 10.1099/ijs.0.63928-0

[35] Přidal A (2002) Effects of three bacteria species on *Bombus terrestris* male larvae under laboratory conditions. Acta Univ Agric et Silvic Mendel Brun 4: 35–46.

[36] Huang WF, Jiang JH, Chen YW, Wang CH (2007) A *Nosema ceranae* isolate from the honey bee *Apis mellifera* . Apidologie 38: 30–37.

[37] Paxton RJ, Klee J, Korpela S, Fries I (2007) *Nosema ceranae* has infected *Apis mellifera* in Europe since at least 1998 and may be more virulent than *Nosema apis* . Apidologie 38: 558–565.

[38] Li J, Chen W, Wu J, Peng W, An J, Schmid-Hempel P, et al (2012) Diversity of *Nosema* associated with bumblebees (*Bombus* spp.) from China. Int J Parasitol 42: 49–61. 10.1016/j.ijpara.2011.10.005 22138016

[39] Fries I, Paxton RJ, Tengö J, Slemenda SB, da Silva AJ, Pieniazek NJ (1999) Morphological and molecular characterization of *Antonospora scoticae* n. gen., n. sp. (Protozoa, microsporidia) a parasite of the communal bee, *Andrena scotica* Perkins, 1916 (Hymenoptera, Andrenidae). Eur J Protistol 35: 183–193.

[40] Cochran WG (1977) Sampling techniques. New York: John Wiley and Sons 448 p.

[41] Boonham N, Smith P, Walsh K, Tame J, Morris J, Spence N, et al (2002) The detection of Tomato spotted wilt virus (TSWV) in individual thrips using real time fluorescent RT-PCR (TaqMan). J Virol Methods 101: 37–48. 11849682 10.1016/s0166-0934(01)00418-9

[42] Evison SEF, Roberts KE, Laurenson L, Pietravalle S, Hui J, Biesmeijer JC, et al (2012) Pervasiveness of parasites in pollinators. PLOS ONE 7: e30641 10.1371/journal.pone.0030641 22347356 PMC3273957

[43] James RR, Skinner JS (2005) PCR diagnostic methods for *Ascosphaera* infection. J Invert Path 90: 98–103. 10.1016/j.jip.2005.08.004 16214164

[44] R Core Team (2014) R: A language and environment for statistical computing. Vienna, Austria: R Foundation for Statistical Computing Available: http://www.R-project.org/. 10.1016/j.jneumeth.2014.06.019

[45] Altschul SF, Gish W, Miller W, Myers EW, Lipman DJ (1990) Basic local alignment search tool. J Mol Biol 215: 403–410. 2231712 10.1016/S0022-2836(05)80360-2

[46] Williamson ECM, Leeming JP, Palmer HM, Steward CG, Warnock D, Marks DI, et al (2000) Diagnosis of invasive aspergillosis in bone marrow transplant recipients by polymerase chain reaction. Brit J Haematol 108: 132–139. 10651736 10.1046/j.1365-2141.2000.01795.x

[47] Visvesvara GS, Leitch GJ, Pieniazek NJ, da Silva AJ, Wallace S, Slemenda SB, et al (1995) Short-term *in vitro* culture and molecular analysis of the microsporidian, *Enterocytozoon bieneusi* . J Euk Microbiol 42: 506–510. 7581324 10.1111/j.1550-7408.1995.tb05896.x

[48] Bakonyi T, Derakhshifar I, Grabensteiner E, Nowotny N (2003) Development and evaluation of PCR assays for the detection of *Paenibacillus larvae* in honey samples: comparison with isolation and biochemical characterization. Appl Environ Microbiol 69: 1504–1510. 10.1128/AEM.69.3.1504-1510.2003 12620836 PMC150092

[49] Cox-Foster DL, Conlan S, Holmes EC, Palacios G, Evans JD, Moran NA, et al (2007) A metagenomic survey of microbes in honey bee colony collapse disorder. Science 318: 283–287. 10.1126/science.1146498 17823314

[50] Baldo L, Dunning Hotopp JC, Jolley KA, Bordenstein SR, Biber SA, Choudhury RR, et al (2006) Multilocus sequence typing system for the endosymbiont *Wolbachia pipientis* . Appl Environmental Microbiol 72: 7098–7110. 10.1128/AEM.00731-06 16936055 PMC1636189

[51] Klinger EG, James RR, Youssef NN, Welker DL (2013) A multi-gene phylogeny provides additional insight into the relationships between several *Ascosphaera* species. J Invertebr Pathol 112: 41–48. 10.1016/j.jip.2012.10.011 23147103

[52] Peterson SW (2008) Phylogenetic analysis of *Aspergillus* species using DNA sequences from four loci. Mycologia 100: 205–226. 18595197 10.3852/mycologia.100.2.205

[53] Maddison WP, Maddison DR (2011) Mesquite: a modular system for evolutionary analysis. Version 2.75. Available: http://mesquiteproject.org.

[54] Stamatakis A (2006) RAxML-VI-HPC: Maximum likelihood-based phylogenetic analyses with thousands of taxa and mixed models. Bioinformatics 22: 2688–2690. 16928733 10.1093/bioinformatics/btl446

[55] Darriba D, Taboada GL, Doallo R, Posada D (2012) jModelTest 2: more models, new heuristics and parallel computing. Nat Methods 9: 772 10.1038/nmeth.2109 22847109 PMC4594756

[56] Akaike H (1974) A new look at the statistical model identification. IEEE T Automat Contr 19: 716–723.

[57] Schwarz G (1978) Estimating the dimension of a model. Ann Stat 6: 461–464.

[58] Minin V, Abdo Z, Joyce P, Sullivan J (2003) Performance-based selection of likelihood models for phylogeny estimation. Syst Biol 52: 674–683. 14530134 10.1080/10635150390235494

[59] Guindon S, Dufayard JF, Lefort V, Anisimova M, Hordijk W, Gascuel O (2010) New algorithms and methods to estimate maximum-likelihood phylogenies: assessing the performance of PhyML 3.0. Syst Biol 59: 307–321. 10.1093/sysbio/syq010 20525638

[60] Ronquist F, Teslenko M, van der Mark P, Ayres DL, Darling A, Höhna S, et al (2012) MrBayes 3.2: efficient Bayesian phylogenetic inference and model choice across a large model space. Syst Biol 61: 539–542. 10.1093/sysbio/sys029 22357727 PMC3329765

[61] Brown JE, Hedtke SM, Lemmon AR, Lemmon EM (2010) When trees grow too long: investigating the causes of highly inaccurate Bayesian branch-length estimates. Syst Biol 59: 145–161. 10.1093/sysbio/syp081 20525627

[62] Rambaut A, Suchard MA, Xie D, Drummond AJ (2014) Tracer, version 1.6. Available: http://beast.bio.ed.ac.uk/Tracer.

[63] Nylander JA, Wilgenbusch JC, Warren DL, Swofford DL (2008) AWTY (are we there yet?): a system for graphical exploration of MCMC convergence in Bayesian phylogenetics. Bioinformatics 24: 581–583. 17766271 10.1093/bioinformatics/btm388

[64] Paradis E, Claude J, Strimmer K (2004) APE: analyses of phylogenetics and evolution in R language. Bioinformatics 20: 289–290. 14734327 10.1093/bioinformatics/btg412

[65] Anderson DL, Gibbs AJ, Gibson NL (1998) Identification and phylogeny of spore-cyst fungi (*Ascosphaera* spp.) using ribosomal DNA sequences. Mycol Res 102: 541–547.

[66] Wynns AA, Jensen AB, Eilenberg J (2013) *Ascosphaera callicarpa*, a new species of bee-loving fungus. PLOS ONE 8: e73419 10.1371/journal.pone.0073419 24086280 PMC3783469

[67] Skou JP (1982) *Ascosphaera asterophora* species nova. Mycotaxon 14: 149–159.

[68] Zayed A, Constantin SA, Packer L (2007) Successful biological invasion despite a severe genetic load. PLOS ONE 2: e868 10.1371/journal.pone.0000868 17848999 PMC1964518

[69] Sheffield CS, Ratti C, Packer L, Griswold T (2011) Leafcutter and mason bees of the genus *Megachile* Latreille (Hymenoptera: Megachilidae) in Canada and Alaska. Can J Arthropod Ident 18: 1–107.

[70] Medler JT (1959) A note on *Megachile centuncularis* (Linn.) in Wisconsin (Hymenoptera: Megachilidae). Can Entomol 91:113–115.

[71] Spiltoir CF (1955) Life cycle of *Ascosphaera apis* . Am J Bo 2: 501–518.

[72] Colla SR, Otterstatter MC, Gegear RJ, Thomson JD (2006) Plight of the bumble bee: pathogen spillover from commercial to wild populations. Biol Cons 129: 461–467.

[73] Charles MVP, Noyal MJ, Easow JM, Ravishankar M (2011) Invasive pulmonary aspergillosis caused by *Aspergillus versicolor* in a patient on mechanical ventilation. Australas Med J 4: 632–634. 10.4066/AMJ.2011.905 23386878 PMC3562921

[74] Kim K, Alker AP, Shuster K, Quirolo C, Harvell CD (2006) Longitudinal study of aspergillosis in sea fan corals. Dis Aquat Organ 69: 95–9. 16703771 10.3354/dao069095

[75] Shin SH, Kim S, Kim JY, Song HY, Cho SJ, Kim DR, et al (2012) Genome sequence of *Paenibacillus terrae* HPL-003, a xylanase-producing bacterium isolated from soil found in forest residue J Bacteriol 194: 1266 10.1128/JB.06668-11 22328761 PMC3294771

[76] Timmusk S, Grantcharova N, Wagner EGH (2005) *Paenibacillus polymyxa* invades plant roots and forms biofilms. Appl Environ Microbiol 71: 7292–7300. 10.1128/AEM.71.11.7292-7300.2005 16269771 PMC1287669

[77] Garcia-Gonzalez S, Genersch E (2013) Honey bee larval peritrophic matrix degradation during infection with *Paenibacillus larvae*, the aetiological agent of American foulbrood of honey bees, is a key step in pathogenesis. Environ Microbiol 15: 2894–2901. 23809335 10.1111/1462-2920.12167

[78] Gerth M, Geißler A, Bleidorn C (2011) Wolbachia infections in bees (Anthophila) and possible implications for DNA barcoding. Syst Biodiv 9: 319–327.

[79] Ravoet J, De Smet L, Meeus I, Smagghe G, Wenseleers T, de Graaf DC (2014) Widespread occurrence of honey bee pathogens in solitary bees. J Invert Path 122: 55–58. 10.1016/j.jip.2014.08.007 25196470

[80] Ladurner E, Bosch J, Kemp WP, Maini S (2005) Assessing delayed and acute toxicity of five formulated fungicides to *Osmia lignaria* Say and *Apis mellifera* . Apidologie 36: 449–460.

[81] Scott-Dupree C, Conroy L, Harris C (2009) Impact of currently used or potentially useful insecticides for canola agroecosystems on *Bombus impatiens* (Hymenoptera: Apidae), *Megachile rotundata* (Hymenoptera: Megachilidae), and *Osmia lignaria* (Hymenoptera: Megachilidae). J Econ Entomol 102: 177–182. 19253634 10.1603/029.102.0125

[82] Di Prisco G, Cavaliere V, Annoscia D, Varricchio P, Caprio E, Nazzi F, et al (2013) Neonicotinoid clothianidin adversely affects insect immunity and promotes replication of a viral pathogen in honey bees. Proc Natl Acad Sci USA 110: 18466–18471. 10.1073/pnas.1314923110 24145453 PMC3831983

[83] Zhang X, He SY, Evans JD, Pettis JS, Yin GF, Chen YP (2012) New evidence that deformed wing virus and black queen cell virus are multi-host pathogens. J Invert Pathol 109: 156–159. 10.1016/j.jip.2011.09.010 22001629

